# Effects of a comprehensive management mode for overweight/obesity based on mobile internet technology and traditional Chinese medicine constitution theory: a randomized controlled trial

**DOI:** 10.3389/fnut.2026.1700808

**Published:** 2026-03-11

**Authors:** Xin Chen, Junyi Duan, Ruxin Duan, Yixing Wang, Hua Jiang

**Affiliations:** 1 Department of Geriatrics, Shanghai East Hospital, Tongji University School of Medicine, Shanghai, China; 2 Department of General Practice, Shanghai East Hospital, Tongji University School of Medicine, Shanghai, China; 3 Department of Acupuncture, Yueyang Hospital of Integrated Traditional Chinese and Western Medicine, Shanghai University of Traditional Chinese Medicine, Shanghai, China; 4 Beijing CapitalBio Technology Company Limited, Beijing, China; 5 Department of Internal Medicine of Traditional Chinese Medicine, Shanghai East Hospital, Tongji University School of Medicine, Shanghai, China

**Keywords:** obesity/overweight management, management mode, mobile health technology, traditional Chinese medicine constitution, gut microbiota

## Abstract

**Background:**

We aimed to investigate the overall effectiveness of a comprehensive management mode for overweight/obesity based on mobile internet technology and TCM constitution theory.

**Methods:**

40 subjects were enrolled and randomly divided into control group (traditional obesity management mode) and intervention group (comprehensive obesity management mode). The intervention period was 8 weeks, and the follow-up period was 1 year. The information of body indicators, blood indicators and gut microbiota indicators at baseline, 8 weeks and 1 year were collected and analyzed, the primary outcome was the effective weight loss (≥5% body weight loss) rate at 1 year.

**Results:**

The average age of 40 subjects was 40.3 ± 9.49 years old, with 18 males accounting for 45%. The effective weight loss rate of intervention group was significantly higher than control group (75% vs. 35%; *p* = 0.025) at 1 year. The body weight (6.88 ± 4.00 vs. 3.08 ± 2.35, *p* = 0.001), BMI(2.38 ± 1.23 vs. 1.10 ± 0.87, *p* = 0.001), body fat rate(BFR; 1.8 ± 0.85 vs. 0.82 ± 0.60, *p* = 0.001), fasting plasma glucose (FPG; 1.08 ± 0.69 vs. 0.49 ± 0.91, *p* = 0.027), total cholesterol (TC; 1.72 ± 0.60 vs. 0.77 ± 0.57, *p* = 0.001), Triglyceride (TG; 0.56 ± 0.52 vs. 0.16 ± 0.22, *p* = 0.003), low density lipoprotein cholesterol (LDL-C; 1.35 ± 0.49 vs. 0.71 ± 0.50, *p* = 0.001), and diastolic blood pressure (DBP; 1.80 ± 1.96 vs. 0.60 ± 1.57, *p* = 0.039) decreased more significant in the intervention group. The gut microbiota analysis showed that after intervention, in the intervention group, at phylum level, the Cyanobacteria abundance increased (*p* = 0.040), while the Euryarchaeota abundance decreased (*p* = 0.045). In addition, the functional abundance of Photosynthesis decreased (*p* = 0.008).

**Conclusion:**

The comprehensive overweight/obesity management mode integrating mHealth with TCM constitution showed more significant long-term weight loss effects. The increase of Cyanobacteria and decrease of Euryarchaeota may be associated with this effect, warranting further investigation.

**Clinical trial registration:**

https://www.chictr.org.cn/showproj.html?proj=43245, Identifier, ChiCTR1900025881.

## 1 Introduction

Disruption of the energy balance due to changes in people’s diets (diets with high calorie foods) and lifestyles (increased sedentary time and decreased physical activity) has led to an increased incidence of overweight and obesity (1) The prevalence of overweight (BMI ≥ 24) and obesity (BMI ≥ 28) among global adults have been 39 and 13%, respectively, (2) Obesity is an important independent risk factor for many chronic diseases (3, 4). Meanwhile obesity also promotes the early onset and exacerbation of chronic diseases, reduces healthy life and total life span, and increases the risk of premature death by 1.45 to 2.76 times. WHO has declared obesity as the biggest chronic health problem for global adults (5, 6). Therefore, it is important to develop effective strategies for the management of obesity to reduce the obesity-related adverse effects.

The accessibility of obesity interventions is very important (5). With the development of mobile Internet technology, and mobile health (mHealth) has become a new term in healthcare. mHealth can reduce the number of visits and increases self-monitoring and engagement, while self-monitoring of diet and exercise is the cornerstone of weight loss interventions (7) and the frequency of self-monitoring is positively associated with weight loss outcomes (7). In recent years, there has been a significant increase in the number of studies using mobile Internet technologies to manage overweight/obesity. Although Internet interventions may achieve significant weight loss, many studies have had difficulty maintaining the effect of weight loss, and the long-term weight loss benefits are not significant compared to traditional “face-to-face” obesity management modes (7–9). A review included 18 studies with 2,703 participants found at 12 months’ follow-up, a smartphone app probably resulted in little to no difference in BMI change (10). Maintaining long-term weight loss is a major challenge in current obesity management. Therefore, there is a need to integrate multiple measures to establish a better weight loss mode.

Traditional Chinese Medicine (TCM) shows certain advantages in managing overweight/obesity. Based on the concept of preventing diseases, TCM is a holistic and comprehensive intervention for overweight/obesity, which can effectively reduce weight and improve blood sugar and blood lipid levels (11). The concept of ‘TCM constitution’ is the relatively stable traits of an individual in terms of morphological structure, physiological function and psychological state under the joint influence of genetic and environmental factors (12). The TCM constitution has been studied since 1970s, and the Standards for Classification and Determination of TCM Constitution were issued by the China Association of Chinese Medicine in 2009, turning it into a uniform and standardized method for determining TCM constitution type. Through the standard constitution scale, the human constitution can be classified into 9 types, with phlegm-dampness constitution being one of them. According to the standard phlegm-dampness constitution scale (Table 1) (12), people with a final score of 40 or more are traditionally known as phlegm-dampness constitution in TCM. Final score = [(Raw Score–Number of Items) / (Number of Items × 4)] × 100. Phlegm-dampness constitution is closely related to obesity (13). Prescription that dissolves phlegm and dispels dampness as the main therapeutic principle can effectively reduce the phlegm-dampness constitution scale score, which is better for the obesity regulation of people with phlegm-dampness constitution (12, 13).

**Table 1:** Standard scale of phlegm-dampness constitution of traditional Chinese medicine.

Experience in the past year	No	Slightly	Sometimes	Often	All the time
(1) Did you feel chest o stomach stuffiness?	1	2	3	4	5
(2) Did your body feel heavy or lethargic?	1	2	3	4	5
(3) Was your stomach/belly flabby?	1	2	3	4	5
(4) Did you have an excessively oily forehead and/ or T-zone?	1	2	3	4	5
(5) Did you have upper eyelid swelling?	1	2	3	4	5
(6) Did your mouth feel sticky?	1	2	3	4	5
(7) Did you have lots of phlegm, especially in your throat?	1	2	3	4	5
(8) Did your tongue have a thick coating?	1	2	3	4	5

Final score = [(Raw Score–Number of Items) / (Number of Items × 4)] × 100.

Comprehensive overweight/obesity management mode based on mobile internet technology and TCM constitution theory might be effective in improving the effect of weight loss. Several previous studies have reported that phlegm-dampness constitution constitutes the highest proportion of the overweight/obesity population and is closely associated with the development of many chronic diseases (14, 15). Weight loss of more than 5% is considered effective weight loss and is the threshold for increased benefit in patients with chronic diseases (14). Many researchers have revealed significant differences in transcriptome expression profiles and metabolome profiles between different constitution obesity people. Obesity individuals with phlegm-dampness constitution exhibited more prominent molecular features associated with metabolic disorders, insulin resistance, inflammatory responses, and oxidative stress (15, 16). A lot of studies supported that the gut microbiota played an important role in the progression of obesity and its complications (17, 18). However, presently, the specific mechanism of improving weight loss by regulating constitution has not been fully elucidated.

The purpose of this study was to evaluate the overall effectiveness of the comprehensive overweight/obesity management mode based on mobile internet technology and TCM constitution compared to the traditional overweight/obesity management mode. The primary outcome was the effective weight loss rate of each group. In addition, we also analyzed the gut microbial characteristics to preliminarily explore the underlying mechanism of comprehensive intervention measures based on TCM constitution.

## 2 Materials and methods

### 2.1 Study design

From July 2019 to October 2019, we established the inclusion and exclusion criteria and enrolled 40 subjects who met these criteria. They were randomly recruited and divided into intervention group (comprehensive intervention based on mobile internet technology and TCM constitution theory) and control group (normal comprehensive intervention). Random numbers were generated using Excel. As this study used an open-label design, subjects were informed of the group assignment at the time of randomization. The subjects could not be blinded because of the intervention method. Inclusion criteria: (1) 18–65 years old; (2) 24 kg/m^2^ ≤ BMI < 40 kg/m^2^; (3) with phlegm-dampness constitution (phlegm-dampness constitution scale final score ≥40); (4) informed consent. Exclusion criteria: (1) patients with severe digestive disease or history of gastrointestinal surgery; (2) patients with serious immune system or blood system diseases, or have other serious diseases; (3) patients who have participated in other weight loss programs in the past 6 months; (4) patients who were pregnant. In addition, according to the Guidelines for the Prevention and Control of Overweight and Obesity in Chinese Adults (19), drug treatment was recommended for patients with BMI ≥ 30 kg/m^2^ or BMI ≥ 28 kg/m^2^ with obesity-related diseases. Those who refused drug treatment were again recommended to adopt drug treatment if their weight was still poorly controlled at the end of the study. The primary outcome was the effective weight loss (≥5% body weight loss) rate at 1 year. We set the sample size at 20 cases per group, the sample size was chosen primarily for feasibility and to generate preliminary effect estimates. This study was conducted in Shanghai East hospital, Tongji University School of Medicine, China. It was approved by the Ethics Committee, Shanghai East Hospital (2019ΥS-054 Amendment 1). This study was registered in Chinese Clinical Trial Registry (ChiCTR1900025881, Registration Date: 2019-09-12).

### 2.2 Comprehensive obesity management mode

**Figure 1:**
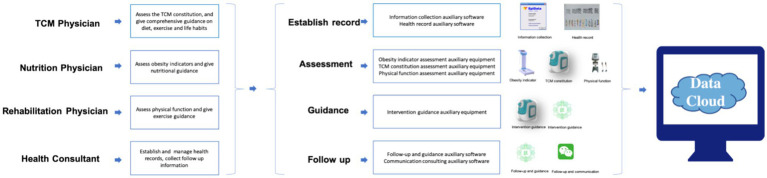
Framework of the comprehensive obesity management mode.

**Figure 2:**
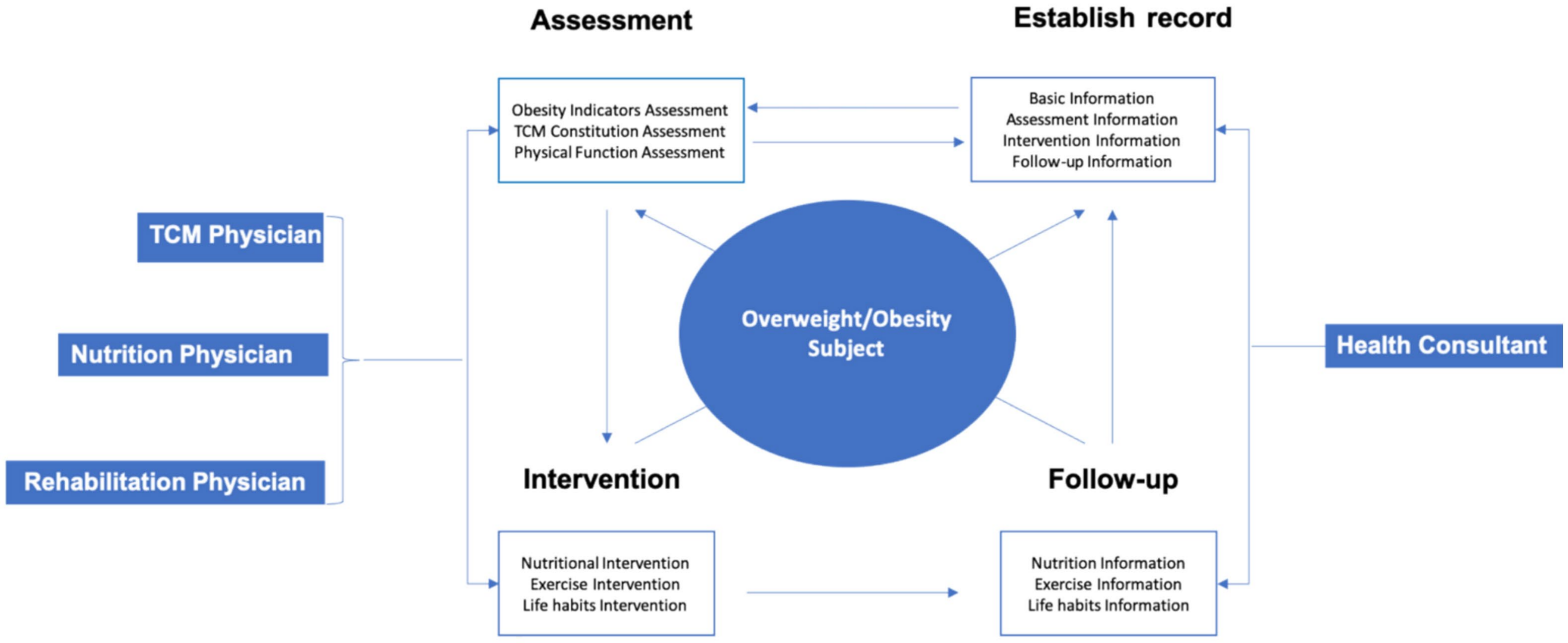
Operation process of the comprehensive obesity management mode.

The comprehensive obesity management mode based on mobile Internet technology and TCM constitution theory was composed of information software equipment and professional physicians (Figure 1). Information software and equipment included: information collection auxiliary software, health record auxiliary software, obesity indicator auxiliary equipment, TCM constitution assessment auxiliary equipment, physical function assessment auxiliary equipment, intervention guideline auxiliary equipment, follow-up and guidance auxiliary equipment and communication consulting auxiliary equipment. Professional physicians: TCM physicians, nutrition physicians, rehabilitation physicians and health consultants. Professional physicians provided information collection, comprehensive assessment, intervention guidance and follow up services for obesity people through these equipment and software. All information was timely uploaded to the data cloud (Figure 2).

### 2.3 Dampness-eliminating grain powder preparation

The powder provided a high-fiber, plant-based nutritional foundation known to benefit metabolism. Its specific composition was structured according to the TCM principle of ‘monarch, minister, assistant, and envoy’ herb pairing, aiming to act synergistically on pathophysiological processes associated with the phlegm-dampness constitution, such as chronic low-grade inflammation, fluid metabolism, and gut homeostasis. This targeted synergy may potentially yield more targeted or enhanced regulatory effects compared to a generic high-fiber diet. Dampness-eliminating grain powder was comprised of 14 ingredients (coix seed powder 9%, lotus seed powder 1.2%, buckwheat powder 7%, oats powder 37.1%, red bean powder 10.5%, white lentil powder 5%, sorghum powder 5%, gravy powder 1.2%, yam powder 2%, poria powder 1.2%, wolfberry powder 0.8%, red date powder 7%, coconut powder powder 10%, algae oil 3%). It was produced by Jiangxi Cereal Food Co., Ltd., Yichun City, Jiangxi Province, China. The preparation process of dampness-eliminating grain powder was as follows:

(1) Pre-treating the qualified herbal raw materials; (2) Grinding and extraction of raw materials according to a specific process; (3) Mixing and blending of various materials according to a fixed ratio; (4) Dispensing into different small packages according to a fixed dosage. Each individual package is 30 g. The dampness-eliminating grain powder were examined for microorganism, heavy metal, and pesticide content.

The ingredients of the dampness-eliminating grain powder were detected according to national testing standards, and the results showed that the dampness-eliminating grain powder contains protein 21%, fat 17%, carbohydrate 19%, dietary fiber 40%, sodium 2%.

### 2.4 Interventions

After evaluating obesity indicator and obesity-related risk factors, TCM physicians assessed the subjects’ TCM constitution and included subjects with phlegm-dampness constitution (phlegm-dampness constitution scale final score ≥40). Nutrition physicians and rehabilitation physicians supplied dietary guidance and exercise guidance for each subject. The nutrition physicians collected information on the subjects’ previous diets and formulated new dietary plans based on the subjects’ eating habits. The daily calories were reduced by 20–30% on the basis of the original diet calories, and the proportion of carbohydrates in the diet was 50–55%, protein was 15–20%, and fat was 25–30%. The rehabilitation physicians assessed the physical abilities of the subjects and developed 30–60 min exercise plans of moderate-intensity physical activity (e.g., jogging, cycling, stair climbing, etc.) according to the subject’s condition. ‘Moderate-intensity’ was objectively defined as exercise eliciting 60–85% of the estimated maximum heart rate (using the formula: 220—age). Subjectively, it corresponded to an exertion level at which participants experienced a noticeable increase in heart rate and breathing but could still talk comfortably. Subjects were asked to follow the dietary and exercise plans for the first 8 weeks.

Dissolving phlegm and dispelling dampness interventions are important part in TCM to manage obese patients with phlegm-dampness constitution. These interventions can be acupuncture, medication, diet, etc. In this study, in order to improve volunteer adherence, we chose to add dampness-eliminating grain powder (Jiangxi Cereal Food Co., Ltd.) to the patients’ diet to regulate phlegm-dampness constitution. For the subjects in intervention group, on the premise that the total calories and nutrients in the diet of the intervention group were the same as those of the control group, 60 g of dampness-eliminating grain powder was added in the daily diet. Subjects in intervention group were asked to recorded their diet and exercise and sent the information to researchers through Wechat, and the researchers conducted follow-up supervision on Wechat every day. After 8 weeks, the subjects were not required to follow a fixed diet and exercise plan. The subjects were followed up on Wechat once or twice a month for 1 year, and face-to-face visits were made at 8 weeks and 1 year.

Subjects in control group were asked to recorded their diet and exercise in a notebook every day and received face-to-face obesity management guidance per week for the first 8 weeks. After 8 weeks, the subjects were not required to follow a fixed diet and exercise plan. They were followed up by phone or face to face once or twice a month for 1 year, and face-to-face visits were made at 8 weeks and 1 year.

### 2.5 Information collection

The intervention period was 8 weeks, and the follow-up period was 1 year. The information at baseline, 8 weeks, and 1 year were collected, and the changes of each indicator at different time points were analyzed to evaluate the effects of the comprehensive intervention measures based on TCM constitution in overweight/obesity subjects. Information included: (1) demographic information (gender, age, education and income), obesity indicator (height, weight, body fat rate), laboratory indicators information [fasting plasma glucose (FPG), total cholesterol (TC), triglyceride (TG), low density lipoprotein cholesterol (LDL-C), high density lipoprotein cholesterol (HDL-C)], systolic blood pressure (SBP), diastolic blood pressure (DBP) and gut microbiota information.

### 2.6 Gut microbiota analysis

#### 2.6.1 DNA extraction and 16S rRNA sequencing

Stool samples were freshly collected from each individual at baseline and 8 weeks, and stored in a − 80 °C refrigerator for freezing. Considering the cost-effectiveness and statistically sound, we randomly selected 10 subjects per group after the sample collections were completed. The 40 fecal samples of these 20 subjects before and after intervention were couriered to Beijing CapitalBio Technology Co. for high-throughput sequencing of the same batch.

The DNA of samples were extracted by hexadecyltrimethylammonium bromide (CTAB) method, then the purity and concentration of DNA were extracted by using agarose gel electrophoresis detection. An appropriate amount of sample DNA was placed in a centrifuge tube, and the sample was diluted to 1 ng/μL with sterile water. PCR amplification was performed using diluted genomic DNA as a template with Barcode primers, Phusion® High-Fidelity PCR Master Mix with GC Buffer (New England Biolabs company), and highly effective high-fidelity enzymes to ensure amplification efficiency and accuracy. The 16S V3-V4 region was amplified with 341F and 806R primers to identify bacterial diversity. TruSeq® DNA PCR-free Sample Preparation Kit was used for library construction. The constructed libraries were quantified by Qubit and Q-PCR. After the qualified libraries were detected, NovaSeq6000 was used for on-machine sequencing.

#### 2.6.2 Sequencing data analysis

The analysis of intestinal microbiota information included three parts: data processing and quality control, basic analysis, and advanced analysis. Data processing and quality control was to obtain high-quality tags that can be used for later analysis through the original sequence stitching, filtering and data evaluation. The basic analysis was to cluster the Operational Taxonomic Units (OTU) of the processed tags, and then performed species annotation and diversity analysis. Diversity analysis mainly evaluated the richness of samples, including *α*-diversity analysis and *β*-diversity analysis. Advanced analysis mainly included significant difference analysis, correlation analysis, function prediction, etc. β-diversity analysis was carried out using the Vegan and GUniFrac packages in R language to compare the similarity of different samples in terms of species diversity. The distance between samples was mainly calculated by Weighted (Bray Curtis and Weighted Unifrac) algorithm and Unweighted (Binary Jaccard and Unweighted Unifrac) algorithm, and the β value between samples was obtained. The unweighted algorithm mainly compared the presence or absence of species. The weighting method considered both the presence and abundance of species. Wilcoxon rank sum test and Kruskal-Wallis test were used to analyze the difference of *α*-diversity index between the two groups. *p* < 0.05 was considered statistically significant.

### 2.7 Statistical analysis

Statistical analysis of clinical information was performed using SPSS 24.0 (SPSS Inc., Chicago, IL, United States) software. Continuous variables were expressed as mean ± standard deviation, t-test was used for normally distributed data, and Wilcoxon rank sum test was used for non-normally distributed data. Categorical variables were expressed as percentages and tested by chi-square. Repeated measures ANOVA was used to analyze the changes in the indicators at baseline, 8 weeks, and 1 year, and *p* < 0.05 was considered statistically significant.

## 3 Results

### 3.1 Clinical characteristics

**Figure 3:**
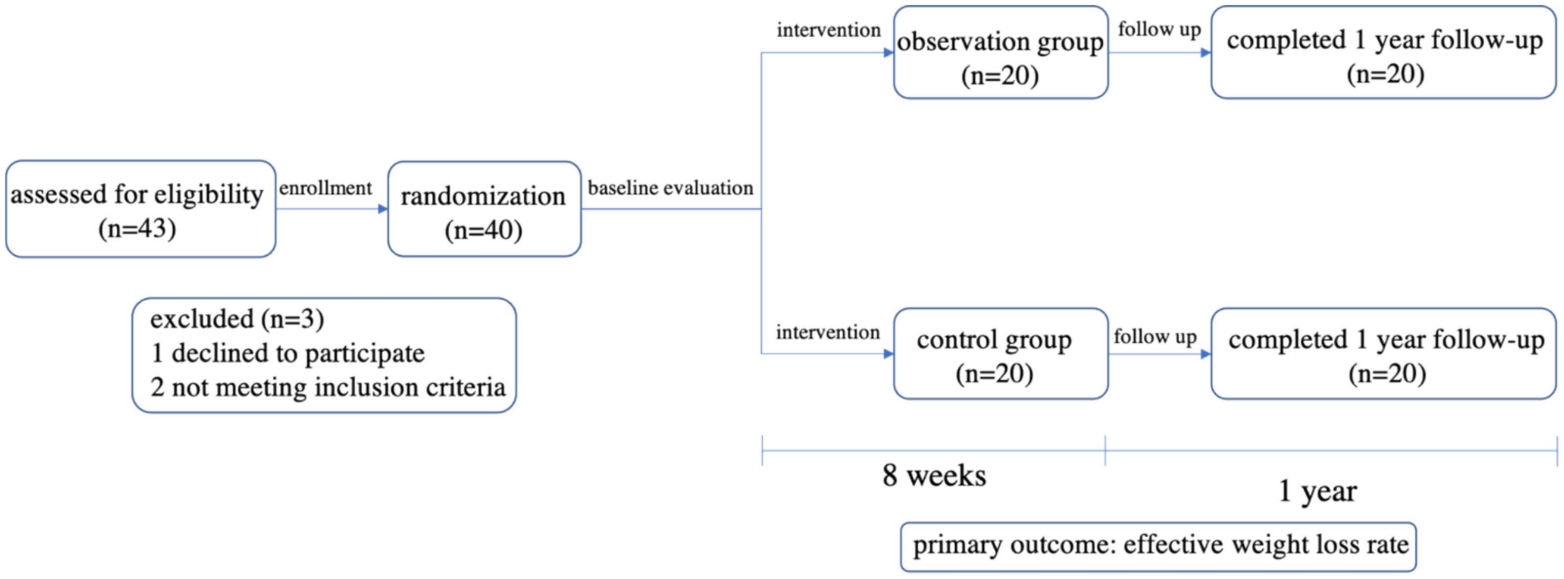
Flowchart of patient enrollment and status.

A total of 40 subjects were enrolled in this study, and all subjects completed the intervention and follow-up without adverse reactions (Figure 3). The mean age of the 40 subjects was 40.3 ± 9.49 years, with 45% males and 55% females. There were no significant differences in gender, age, education, height, weight, BMI, body fat rate, FPG, TC, TG, LDL-C, HDL-C, SBP and DBP between the two groups (*p* > 0.05; Table 2).

**Table 2:** Baseline information of all subjects.

Variables	Total (*n* = 40)	Control group (*n* = 20)	Intervention group (*n* = 20)	*p* value
Sex				1.000
Male, n (%)	18(45)	9(45)	9(45)	
Female, n (%)	22(55)	11(55)	11(55)	
Age (year)	40.30 ± 9.49	42.50 ± 9.56	38.10 ± 9.12	0.145
Education (year)				0.597
≤12 (senior high school), n (%)	8 (20)	5 (25)	3 (15)	
13-16 (junior college/undergraduate), n (%)	21 (52.5)	12 (60)	9 (75)	
≥17 (postgraduate), n (%)	10 (25)	3 (15)	7 (35)	
Height (cm)	166.87 ± 8.03	166.11 ± 8.58	167.63 ± 7.59	0.558
Weight (kg)	80.30 ± 15.59	79.08 ± 14.72	81.53 ± 16.71	0.625
BMI (kg/m^2^)	28.64 ± 3.98	28.51 ± 3.82	28.77 ± 4.23	0.834
BFR (%)	30.76 ± 4.53	29.96 ± 4.47	31.56 ± 4.56	0.271
FPG (mmol /L)	5.92 ± 1.60	6.03 ± 1.84	5.81 ± 1.36	0.661
TC (mmol /L)	5.04 ± 1.02	4.99 ± 1.07	5.08 ± 1.00	0.783
TG (mmol /L)	1.67 ± 0.84	1.62 ± 0.91	1.72 ± 0.79	0.705
LDL-C (mmol /L)	3.11 ± 0.87	3.06 ± 0.98	3.17 ± 0.77	0.689
HDL-C (mmol /L)	1.24 ± 0.29	1.20 ± 0.24	1.27 ± 0.34	0.433
SBP (mmHg)	135.20 ± 11.91	134.85 ± 11.71	135.55 ± 12.40	0.855
DBP (mmHg)	81.00 ± 7.05	81.30 ± 7.95	80.70 ± 6.21	0.792

BMI, Body Mass Index; BFR, Body Fat Rate; FPG, Fasting Plasma Glucose; TC, Total Cholesterol; TG, Triglyceride; LDL-C, Low Density Lipoprotein Cholesterol; HDL-C, High Density Lipoprotein Cholesterol; SBP, Systolic blood pressure; DBP, Diastolic Blood Pressure.

After 8 weeks of intervention, the effective weight loss rate of the control group was 75% and that of the intervention group was 90%, but there was no significant difference between the two groups (*p* = 0.204). Compared with the baseline level, there were no significant differences in the decrease values of body weight, BMI, body fat rate, HDL-C, SBP and DBP between the two groups (*p* > 0.05). The decrease values of FPG, TC, TG, and LDL-C in the intervention group were significantly greater than those in the control group (*p* < 0.05; Table 3).

**Table 3:** Changes of obesity indicators and laboratory indicators after 8 weeks of all subjects.

Variables	Total (*n* = 40)	Control group (*n* = 20)	Intervention group (*n* = 20)	*p*value
Effective weight loss rate, n(%)	33(82)	15(75)	18(90)	0.204
Weight decreased value	5.11 ± 1.60	4.89 ± 1.79	5.34 ± 1.40	0.381
BMI decreased value	1.81 ± 0.50	1.74 ± 0.55	1.89 ± 0.44	0.356
BFR decreased value	1.35 ± 0.31	1.30 ± 0.36	1.40 ± 0.24	0.283
FPG decreased value	0.86 ± 0.68	0.65 ± 0.67	1.07 ± 0.63	0.049
TC decreased value	1.51 ± 0.17	1.42 ± 0.19	1.60 ± 0.07	0.001
TG decreased value	0.47 ± 0.47	0.26 ± 0.43	0.68 ± 0.41	0.003
LDL-C decreased value	1.20 ± 0.30	1.07 ± 0.33	1.34 ± 0.19	0.003
HDL-C decreased value	−0.01 ± 0.06	−0.01 ± 0.05	−0.01 ± 0.07	0.819
SBP decreased value	2.25 ± 2.63	2.3 ± 2.25	2.28 ± 2.42	0.949
DBP decreased value	1.1 ± 1.21	1.2 ± 1.2	1.15 ± 1.19	0.749

BMI, Body Mass Index; BFR, Body Fat Rate; FPG, Fasting Plasma Glucose; TC, Total Cholesterol; TG, Triglyceride; LDL-C, Low Density Lipoprotein Cholesterol; HDL-C, High Density Lipoprotein Cholesterol; SBP, Systolic blood pressure; DBP, Diastolic Blood Pressure.

At 1-year follow-up, the effective weight loss rate of the control group was 35%, and the effective weight loss rate of the intervention group was 75%, with significant difference between the two groups (*p* = 0.025). Meanwhile, compared with the baseline level, the decrease values of body weight, BMI, body fat rate, FPG, TC, TG, LDL-C, and DBP in the intervention group were significantly higher than those in the control group (*p* < 0.05; Table 4).

**Table 4:** Changes of obesity indicators and laboratory indicators after 1 year of all subjects.

Variables	Total (*n* = 40)	Control group (*n* = 20)	Intervention group (*n* = 20)	*p*value
Effective weight loss rate, n(%)	22(55)	7(35)	15(75)	0.025
Weight decreased value	4.98 ± 3.77	3.08 ± 2.35	6.88 ± 4.00	0.001
BMI decreased value	1.74 ± 1.23	1.10 ± 0.87	2.38 ± 1.23	0.001
BFR decreased value	1.31 ± 0.88	0.82 ± 0.60	1.8 ± 0.85	0.001
FPG decreased value	0.79 ± 0.85	0.49 ± 0.91	1.08 ± 0.69	0.027
TC decreased value	1.24 ± 0.75	0.77 ± 0.57	1.72 ± 0.60	0.001
TG decreased value	0.36 ± 0.44	0.16 ± 0.22	0.56 ± 0.52	0.003
LDL-C decreased value	1.03 ± 0.59	0.71 ± 0.50	1.35 ± 0.49	0.001
HDL-C decreased value	−0.03 ± 0.05	−0.03 ± 0.04	−0.04 ± 0.06	0.512
SBP decreased value	2.15 ± 3.52	1.30 ± 3.33	3.00 ± 3.58	0.128
DBP decreased value	1.20 ± 1.86	0.60 ± 1.57	1.80 ± 1.96	0.039

BMI, Body Mass Index; BFR, Body Fat Rate; FPG, Fasting Plasma Glucose; TC, Total Cholesterol; TG, Triglyceride; LDL-C, Low Density Lipoprotein Cholesterol; HDL-C, High Density Lipoprotein Cholesterol; SBP, Systolic blood pressure; DBP, Diastolic Blood Pressure.

Repeated Measures ANOVA showed that the levels of body weight, BMI, body fat rate, FPG, TC, TG, LDL-C, HDL-C, SBP and DBP in the two groups decreased with time (*p* < 0.05). HDL-C increased with time (*p* < 0.05). There were interaction effects between intervention and time. The body weight, BMI, body fat rate, TC, TG, LDL-C, and DBP of the two groups decreased differently over time, and the decrease was more obvious in the intervention group (*p* < 0.05). In the Greenhouse–Geisser analysis, the decrease in FPG levels with time was different in the two groups, and the decrease was greater in the intervention group (Table 5).

**Table 5:** Changes of all indicators with time.

Variables	Group	Baseline	8 week	1 year	*P*_WSC_	*P*_GG_
Weight	Control group	79.08 ± 14.72	74.19 ± 13.33	76.00 ± 14.15	*P*_T_ = 0.001	*P*_T_ = 0.001
	Intervention group	81.53 ± 16.71	76.2 ± 15.58	74.66 ± 14.28	*P*_T*G_ = 0.001	*P*_T*G_ = 0.001
	Total	80.3 ± 15.59	75.19 ± 14.35	75.33 ± 14.05		
BMI	Control group	28.51 ± 3.82	26.77 ± 3.51	27.4 ± 3.72	*P*_T_ = 0.001	*P*_T_ = 0.001
	Intervention group	28.77 ± 4.23	26.89 ± 3.9	26.4 ± 3.75	*P*_T*G_ = 0.001	*P*_T*G_ = 0.001
	Total	28.64 ± 3.98	26.83 ± 3.66	26.9 ± 3.72		
BFR	Control group	29.96 ± 4.47	28.67 ± 4.54	29.14 ± 4.30	*P*_T_ = 0.001	*P*_T_ = 0.001
	Intervention group	31.56 ± 4.56	30.16 ± 4.50	29.76 ± 4.81	*P*_T*G_ = 0.001	*P*_T*G_ = 0.001
	Total	30.76 ± 4.53	29.41 ± 4.53	29.45 ± 4.51		
FPG	Control group	6.03 ± 1.84	5.38 ± 1.22	5.54 ± 1.22	*P*_T_ = 0.001	*P*_T_ = 0.001
	Intervention group	5.81 ± 1.36	4.74 ± 0.90	4.73 ± 0.84	*P*_T*G_ = 0.091	*P*_T*G_ = 0.029
	Total	5.92 ± 1.60	5.06 ± 1.11	5.14 ± 1.11		
TC	Control group	4.99 ± 1.07	3.58 ± 0.98	4.23 ± 1.20	*P*_T_ = 0.001	*P*_T_ = 0.001
	Intervention group	5.08 ± 1.00	3.49 ± 1.00	3.37 ± 0.82	*P*_T*G_ = 0.001	*P*_T*G_ = 0.001
	Total	5.04 ± 1.02	3.53 ± 0.97	3.80 ± 1.11		
TG	Control group	1.62 ± 0.91	1.36 ± 0.57	1.46 ± 0.85	*P*_T_ = 0.001	*P*_T_ = 0.001
	Intervention group	1.72 ± 0.79	1.04 ± 0.42	1.15 ± 0.36	*P*_T*G_ = 0.007	*P*_T*G_ = 0.001
	Total	1.67 ± 0.84	1.20 ± 0.52	1.31 ± 0.66		
LDL	Control group	3.06 ± 0.98	1.99 ± 0.81	2.35 ± 0.97	*P*_T_ = 0.001	*P*_T_ = 0.001
	Intervention group	3.17 ± 0.77	1.83 ± 0.66	1.81 ± 0.65	*P*_T*G_ = 0.001	*P*_T*G_ = 0.001
	Total	3.11 ± 0.87	1.91 ± 0.73	2.08 ± 0.86		
HDL	Control group	1.20 ± 0.24	1.21 ± 0.22	1.23 ± 0.22	*P*_T_ = 0.001	*P*_T_ = 0.006
	Intervention group	1.27 ± 0.34	1.28 ± 0.35	1.31 ± 0.35	*P*_T*G_ = 0.762	*P*_T*G_ = 0.699
	Total	1.24 ± 0.29	1.24 ± 0.29	1.27 ± 0.29		
SBP	Control group	134.85 ± 11.71	132.6 ± 9.70	133.55 ± 9.81	*P*_T_ = 0.001	*P*_T_ = 0.001
	Intervention group	135.55 ± 12.40	133.25 ± 11.65	132.55 ± 12.1	*P*_T*G_ = 0.082	*P*_T*G_ = 0.109
	Total	135.2 ± 11.91	132.92 ± 10.59	133.05 ± 10.88		
DBP	Control group	81.3 ± 7.95	80.2 ± 7.47	80.7 ± 7.37	*P*_T_ = 0.001	*P*_T_ = 0.001
	Intervention group	80.7 ± 6.21	79.5 ± 5.89	78.9 ± 6.57	*P*_T*G_ = 0.046	*P*_T*G_ = 0.028
	Total	81.00 ± 7.05	79.85 ± 6.65	79.8 ± 6.95		

BMI, Body Mass Index; BFR, Body Fat Rate; FPG, Fasting Plasma Glucose; TC, Total Cholesterol; TG, Triglyceride; LDL-C, Low Density Lipoprotein Cholesterol; HDL-C, High Density Lipoprotein Cholesterol; SBP, Systolic blood pressure; DBP, Diastolic Blood Pressure.

### 3.2 Gut microbiota analysis

A total of 2,919,050 original sequence was obtained, and 2,904,084 sequence was obtained after cutting and filtering. The average sequence length was 415.31 bp. There was no significant difference in the average sequence length between two groups (*p* > 0.05). The sequencing coverage of each group was above 97%, indicating that the tested sequences accounted for a high percentage of the total sequences, and the results could be used for further in-depth analysis (Supplementary Table S1).

#### 3.2.1 OTU cluster analysis

A total of 1835 Operational Taxonomic Units (OUT) were obtained from 40 samples. The total number of microflora obtained was Domain: 2, Phylum: 23, Class: 36, Order: 73, Family: 124, Genus: 264, Species: 311 (Supplementary Figure S1). Case_T1 represented the intervention group before intervention, including 1,125 OTUS. Case_T2 represented the intervention group after intervention, including 1,038 OTUS. Control_T1 represented the control group before intervention, including 1,171 OTUS. Control_T2 represented the control group after intervention, including 1,149 OTUS. Panel A: the distribution of OTUs of all samples. Panel B: the distribution of OTUs in the intervention group. Panel C: the distribution of OTUs in the control group.

#### 3.2.2 Gut microbiota alpha diversity

The *α* diversity could be expressed by many coefficients. For example, Shannon, Simpson, Ace and Chao index could be used to express the α diversity. The α diversity was positively correlated with Shannon, Ace and Chao index, and negatively correlated with Simpson index. The Shannon index and Ace index in two groups showed an increasing trend after intervention, and the Simpson index showed a decreasing trend, but there was no statistical difference (*p* > 0.05; Supplementary Table S2; Supplementary Figure S2).

#### 3.2.3 Gut microbiota beta diversity

Although there was a trend of changes in the microbiota characteristics both in the intervention group and the control group after the intervention, there was no significant statistical difference in principal co-ordinates analysis(PCoA; *p* > 0.05; Supplementary Figure S3). According to the *β* diversity distance matrix, the R package pheatmap was used to show the cluster heatmap. The data value was represented by the color depth, red indicated the highest thermal value, and blue indicated the lowest thermal value. The degree of color similarity could reflect the similarity between multiple samples. In this study, we found the similarity between the samples of the intervention group and the control group before and after the intervention was high, with no significant difference (Supplementary Figure S4).

Line Discriminant Analysis Effect Size (LEfSe) analysis could detect species with significant differences in abundance between different groups. The Kruskal-Wallis rank sum test was used to detect species with significant differences in abundance between different groups (Tables 6, 7). Linear Discriminant Analysis (LDA) was used to assess the impact of significantly different species (LDA Score; Supplementary Figures S5, S6). The figure showed species with an LDA Score greater than 2.0, and the length of the bar graph represents the magnitude of the impact of different species.

**Table 6:** Changes of gut microbiota abundance of the intervention group after intervention.

Gut microbiota	Intervention group before intervention	Intervention group after intervention	*p* value
Phylum			
Cyanobacteria	0.399%	1.529%	0.040
Euryarchaeota	0.633%	0.017%	0.045
Class			
Oxyphotobacteria	0.155%	1.383%	0.004
Methanobacteria	0.633%	0.017%	0.045
Order			
Propionibacteriales	3.305%	13.652%	0.013
Chloroplast	0.155%	1.383%	0.004
Methanobacteriales	0.633%	0.017%	0.045
Family			
Propionibacteriaceae	3.289%	13.619%	0.013
Neisseriaceae	0.291%	1.982%	0.012
Uncultured	2.026%	0.036%	0.035
Methanobacteriaceae	0.633%	0.017%	0.045
Genus			
Anaerococcus	0.191%	0.793%	0.047
Negativibacillus	3.209%	1.278%	0.034
Geobacillus	0	1.246%	0.030
Peptoniphilus	0.408%	3.580%	0.013
Methanobrevibacter	0.517%	0	0.013
Cutibacterium	3.153%	13.619%	0.011
Desulfovibrio	5.288%	1.432%	0.011
Lawsonella	0	1.464%	<0.001
Species			
Intestinimonas_butyriciproducens	0.143%	0.525%	0.037
Geobacillus_stearothermophilus	0	1.246%	0.030
Bifidobacterium_animalis	0.152%	0.782%	0.014
Cutibacterium_acnes	2.943%	13.209%	0.013
uncultured_Methanobrevibacter	0.517%	0	0.013
Desulfovibrio_piger	5.152%	1.347%	0.011

**Table 7:** Changes of gut microbiota abundance of the control group after intervention.

Gut microbiota	Control group before intervention	Control group after intervention	*p* value
Family			
Clostridiaceae_1	10.474%	3.733%	0.034
Genus			
Clostridium_sensu_stricto_1	10.347%	3.621%	0.049
Species			
Lactococcus_garvieae	0.330%	0.012%	0.035

#### 3.2.4 Function prediction analysis

**Figure 4:**
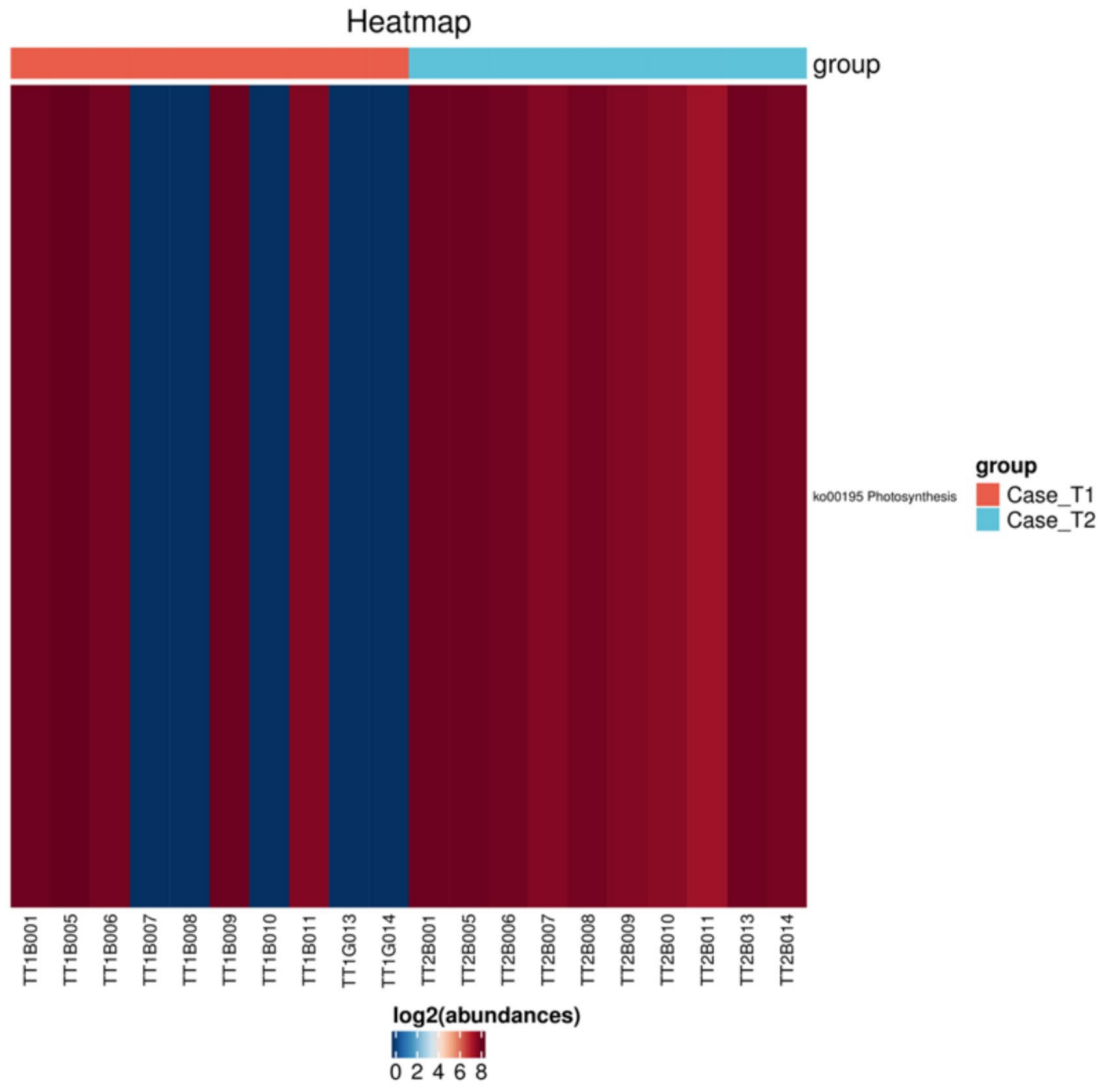
KEGG pathway clustering heat map.

Using PICRUSt2 (Phylogenetic Investigation of Communities by Reconstruction of Unobserved States) for functional prediction of 16S rDNA gene sequencing data, R package limma was used to test the significant differences between the two groups, and then the KEGG (Kyoto Encyclopedia of Genes and Genomes, KEGG) metabolic pathway differences were analyzed. With *p* < 0.05 and fold change>2 as screening conditions, the differences and changes of functional genes in metabolic pathways and protein functions of microbial communities between different groups could be observed. Comparing the functional abundance of gut microbiota genes in the intervention group and the control group before and after intervention, the results showed that the functional abundance of Photosynthesis in the intervention group decreased after intervention (*p* = 0.008; Figure 4). The functional abundance of gut microbiota genes in the control group was not significantly changed after intervention (*p* > 0.05).

## 4 Discussion

This study demonstrated that the comprehensive management mode integrating mHealth with TCM constitution theory yielded superior and sustained weight loss and metabolic benefits at 1 year compared to traditional management mode. This suggested that the integration strategy as a key element, beyond the contribution of any single component. We now consider the advantages of this integrated mode, drawing on our clinical and gut microbiota results to explore its potential mechanisms.

We found no difference in the effective weight loss rate at 8 weeks between two groups, but the effective weight loss rate at 1 year was significantly higher in the intervention group. This is consistent with previous research findings on remote obesity management (20, 21). Some researchers have pointed out that the health benefit of those who can effectively lose weight (weight loss rate ≥5%) was significantly higher than that of those who moderately lose weight (weight loss<5%) (22), so the health benefit of the intervention group was higher than that of the control group. However, some studies have also reported that Internet-based remote obesity management mode have no significant advantage over traditional models in terms of maintaining weight loss results (23). Lugones-Sanchez C et al. explored the weight loss effectiveness of a multicomponent mobile health intervention and found benefits in weight loss and time spent in light physical activity compared with control group, but there was no difference at the 12-month follow-up (24). Delia S West reported Individuals receiving Internet + Incentives interventions were significantly more likely to maintain weight loss ≥ 5% at Month 12 and remain weight stable (25). In addition, previous studies also reported inconsistent findings regarding the effects of internet-based remote interventions on improving parameters of obesity-related chronic diseases (26–31). But the results of this study showed that the comprehensive overweight/obesity management mode had more significant effects on obesity indicators, blood pressure, blood glucose and blood lipid levels, indicating that it had a more significant effect on reducing the risk of chronic diseases in overweight and obesity adults.

The reason for the differences in results may be related to the fact that many remote obesity management models rely primarily on the voluntary participation of subjects and lack communication and supervision of subjects by managers, resulting in a low level of completion of subjects’ obesity management plans. In addition, many remote obesity management modes failed to personalize the program based on the subject’s specific information. The comprehensive overweight/obesity management mode in this study emphasized both remote communication and supervision of subjects by the mode managers during the process to ensure the degree of completion, and the combination of TCM composition theory to provide subjects with personalized obesity management interventions that facilitated the long-term maintenance of management effects. In the future, a more intelligent weight loss mode can be constructed on the basis of this study with the help of big data and AI technology, which can automatically generate a personalized weight reduction plan according to the patient’s condition. This can further enhance the generalizability of the model.

We included subjects with phlegm-dampness constitution, besides the exercise and diet plan, they were also given 60 g of dampness-eliminating grain powder per day. In animal studies, some researchers found that the number of gut microbiota in mice at the door level of Cyanobacteria increased significantly after the dampness-eliminating intervention was given to mice (32), and this was consistent with the results of this study. In addition, the poria cocos in grain powder could reduce intestinal mucosal inflammation, inhibit the growth of harmful bacteria and promote the growth of beneficial bacteria (33). Another animal study confirmed that poria cocos could improve the level of glues and lipid by regulating gut microbiota, and had the potential to prevent and treat hyperglycemia and hyperlipidemia (34). In clinical studies, poria cocos has also been proved to reduce oxidative stress, regulate hormone metabolism, improve gut microbiota and reshape DNA methylation (35). Gordon euryale seed in grain powder also has significant hypoglycemic and lipid-regulating effects. Previous studies used Gordon euryale seed powder to inculcate diabetic mice for 4 weeks, and found that the body weight, the levels of blood glucose, TC, TG and LDL-C have decreased, and the levels of HDL-C have increased, and the levels of inflammatory cytokines such as Tumor Necrosis Factor-*α* (TNF-α) and Interleukin-6 (IL-6) decreased significantly. Moreover, the number of probiotics (bifidobacteria, enterococcus, etc.) in gut microbiota of mice increased significantly, and the number of harmful bacteria (clostridium, etc.) decreased significantly (36). Dampness-eliminating grain powder, as a part of comprehensive management mode for overweight /obesity people with phlegm-dampness constitution, was important to improve the obesity indicators, blood pressure, blood glucose and blood lipid. However, the degree of effect and specific mechanisms still need to be further verified.

Changes in the composition and function of the gut microbiota are associated with metabolic and inflammatory diseases (37). The number of OTU decreased in both groups after intervention, which might be caused by diet control (38, 39). The significant gut microbiota alterations observed specifically in the intervention group, notably the increase in Cyanobacteria and decrease in Euryarchaeota, may provide a preliminary biological lens into the mechanism. LEfSe analysis revealed more substantial gut microbiota alterations in the intervention group. At the phylum level, the intervention led to a significant increase in Cyanobacteria and a significant decrease in Euryarchaeota. Cyanobacteria possess properties beneficial for cardiometabolic health, including anti-inflammatory and insulin-sensitizing effects, partly attributed to compounds like phycocyanin which can inhibit NADPH-driven oxidative stress in adipocytes (40, 41). Consequently, Cyanobacteria supplementation has shown therapeutic promise in conditions like dyslipidemia and hypertension (42, 43). Conversely, methanogens within Euryarchaeota are implicated in obesity progression and are risk factors for gastrointestinal disorders (44), potentially through involvement in chronic inflammation (45). However, their role remains complex, as some studies found no abundance difference in obesity but an increase in anorexia nervosa (46), warranting further large-scale studies. In the function prediction analysis, a predicted decrease in the Photosynthesis pathway was observed post-intervention. As both Cyanobacteria and Euryarchaeota can contribute to this function, the net decrease suggests the reduction in Euryarchaeota had a dominant effect over the increase in Cyanobacteria. The microbiota changes are not merely associated with weight loss in general, but may be specifically tied to the TCM constitution-modulating component of our comprehensive mode.

The superior long-term efficacy of our comprehensive mode likely stems from the synergistic integration of its core components, rather than the effect of any single element. First, TCM constitution theory provided the foundation for personalized targeting. By focusing specifically on individuals with phlegm-dampness constitution, the dampness-eliminating grain powder served as a constitution-tailored tool to address this ‘root’ condition, potentially making the body more responsive to lifestyle changes. Second, mobile internet technology enabled the practical delivery and reinforcement of this personalized approach. The platform facilitated not only daily monitoring and feedback on diet/exercise but also ensured consistent adherence to the constitution-modulating dietary supplement. This created a closed-loop system where real-time behavioral support amplified the effects of biological modulation. This synergy between TCM constitution regulation and mHealth might explain our mode’s success in maintaining weight loss effects.

We observed that there might be a connection between the results of the gut microbiota analysis at 8 weeks and the clinical outcomes at 1 year of follow-up. We thought the comprehensive management mode, particularly the incorporation of TCM constitution-based dietary modulation, induced early and potentially pivotal alterations in the gut microbial ecology. The significant increase in Cyanobacteria and decrease in Euryarchaeota at the phylum level, along with the downregulation of the Photosynthesis functional pathway observed at 8 weeks, may represent a critical biological shift initiated by the intervention. These early changes in the gut microbiota could have created a more favorable intestinal environment, potentially involving reduced inflammation, improved metabolic signaling, or enhanced barrier function that facilitated the maintenance of healthier lifestyle habits (diet and exercise) and metabolic improvements over the subsequent months, even after the intensive 8-week plan concluded. The remote supervision and support via mobile internet technology likely synergized with this altered microbial state to promote sustained adherence. Thus, the 8-week microbial shifts may be interpreted as an early mechanistic biomarker and a contributing factor enabling the long-term sustainability of weight loss and metabolic benefits. Future studies with serial microbiota sampling throughout the intervention and follow-up period are needed to dynamically trace this relationship and validate the role of early microbial remodeling in sustaining long-term health effects.

A central consideration in interpreting our findings is the multi-component design of the intervention in the intervention group. This study was conceived as a pragmatic pilot trial to assess the combined effect of a novel management package, rather than to isolate the contribution of its individual elements (e.g., the TCM grain powder vs. intensified mHealth supervision). The rationale for this integration is theorized synergy: the mobile platform provides the structure for sustained delivery and monitoring of the constitution-tailored dietary intervention (the grain powder) and personalized lifestyle plans, while the TCM-based modulation may improve the internal metabolic milieu, potentially enhancing adherence and physiological responsiveness to lifestyle changes. Thus, the observed superior long-term outcomes should be attributed to the integrated package as a whole. This design reflects a real-world approach to complex behavioral interventions, where combined strategies are often deployed. To disentangle the specific effects of each component, future research should employ factorial designs (e.g., comparing standard care, mHealth-alone, TCM modulation-alone, and the full package) to identify active ingredients and their potential interactions.

Our study has some limitations. The sample size was small, and the subjects were all phlegm-dampness constitution, which limits the statistical power for subgroup analyses and increases the vulnerability of the findings to random error. The results of this study may inform the design of a future definitive large-scale trial, and it is necessary to conduct a larger sample study to further verify the effect of the comprehensive obesity management mode. Secondly, we only collected stool samples from subjects at baseline and 8 weeks, without dynamic observation of gut microbiota, and the follow-up period was only 1 year, which could not assess the maintenance of weight loss effects in a longer term. Future studies should increase the time points of sample collection, dynamically observe the changes in gut microbiota, extend the follow-up period. Thirdly, the observed superior long-term outcomes should be attributed to the integrated package as a whole. This design reflects a real-world approach to complex behavioral interventions, where combined strategies are often deployed. To disentangle the specific effects of each component, future research should employ factorial designs (e.g., comparing standard care, mHealth-alone, TCM modulation-alone, and the full package) to identify active ingredients and their potential interactions. Finally, this study only initially explored the mechanism of action of the comprehensive obesity management mode from the perspective of gut microbiota, and other indicators (such as inflammatory factors, neuroendocrine factors or hormones) should be added later to analyze the mechanism of this mode more comprehensively.

In conclusion, compared with the traditional obesity management mode, the comprehensive overweight/obesity management mode had more significant long-term weight loss effects. The increase of Cyanobacteria and the decrease of Euryarchaeota leading to the functional abundance decrease in Photosynthesis, might be a potential target for maintaining weight loss effect and regulating the level of blood pressure, blood glucose, and blood lipids.

## Data Availability

The original contributions presented in the study are included in the article/Supplementary material, further inquiries can be directed to the corresponding authors.
