# Supplementary material for: Effects of a comprehensive management mode for overweight/obesity based on mobile internet technology and traditional Chinese medicine constitution theory: a randomized controlled trial
Source: Front Nutr. 2026 Mar 11;13:1700808. doi: 10.3389/fnut.2026.1700808 (PMC13013377; doi:10.3389/fnut.2026.1700808)
Supplement: Supplementary file 1 [file Supplementary_file_1.docx]

**Supplementary Table S1. Sequence information of gut microbiota in each group**

| Group | Average number of sequences | Average sequence length（bp） | Sequencing coverage ratio（%） |
| --- | --- | --- | --- |
| Control group before intervention | 77102.10±10325.52 | 416.40±3.15 | 98.27±0.54 |
| Control group after intervention | 79231.00±7695.82 | 416.41±3.30 | 98.26±0.51 |
| Intervention group before intervention | 71023.30±5114.30 | 417.49±0.95 | 97.81±0.28 |
| Intervention group after intervention | 63052.00±5547.39 | 417.26±2.13 | 97.46±0.36 |

**Supplementary Table S2. α diversity index information**

| Group | Shannon | Simpson | Ace | Chao |
| --- | --- | --- | --- | --- |
| Control group before intervention | 3.39±0.37 | 0.10±0.07 | 461.59±123.07 | 467.03±135.15 |
| Control group after intervention | 3.43±0.35 | 0.08±0.03 | 470.38±78.61 | 441.08±65.02 |
| Intervention group before intervention | 3.27±0.42 | 0.11±0.05 | 475.68±98.47 | 466.98±95.29 |
| Intervention group after intervention | 3.35±0.36 | 0.10±0.04 | 504.66±91.49 | 494.02±90.16 |

**Supplementary Figure S1. OTU distribution Venn diagram**

A: The OUT of 4 groups, B: the OTU of intervention group, C: the OUT of control group


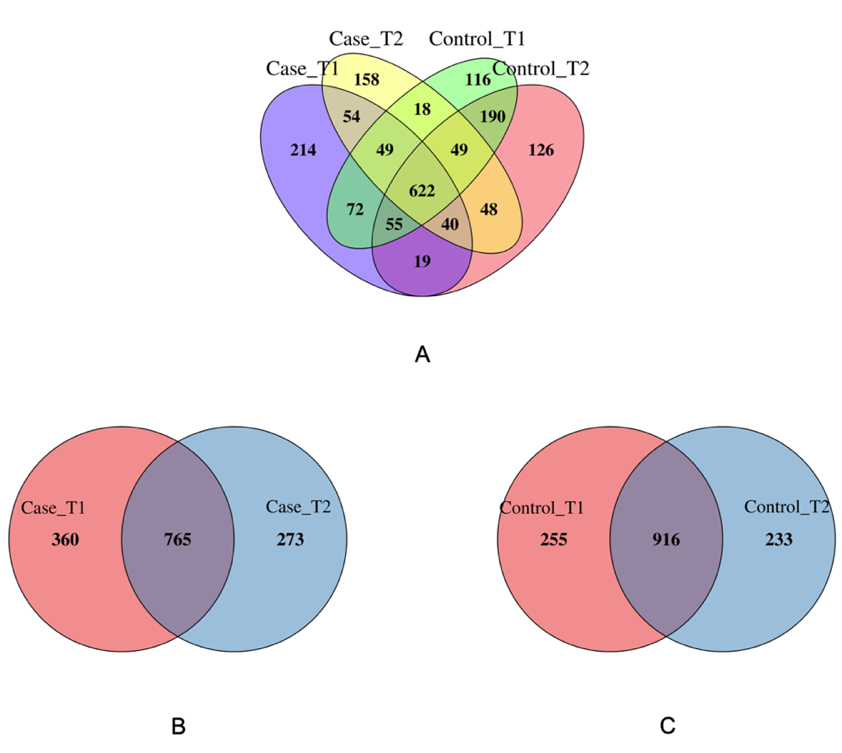


**Supplementary Figure S2.** α diversity index combination

A: Simpson index of each group, B: Shannon index of each group, C: Ace index of each group, D: Chao index of each group


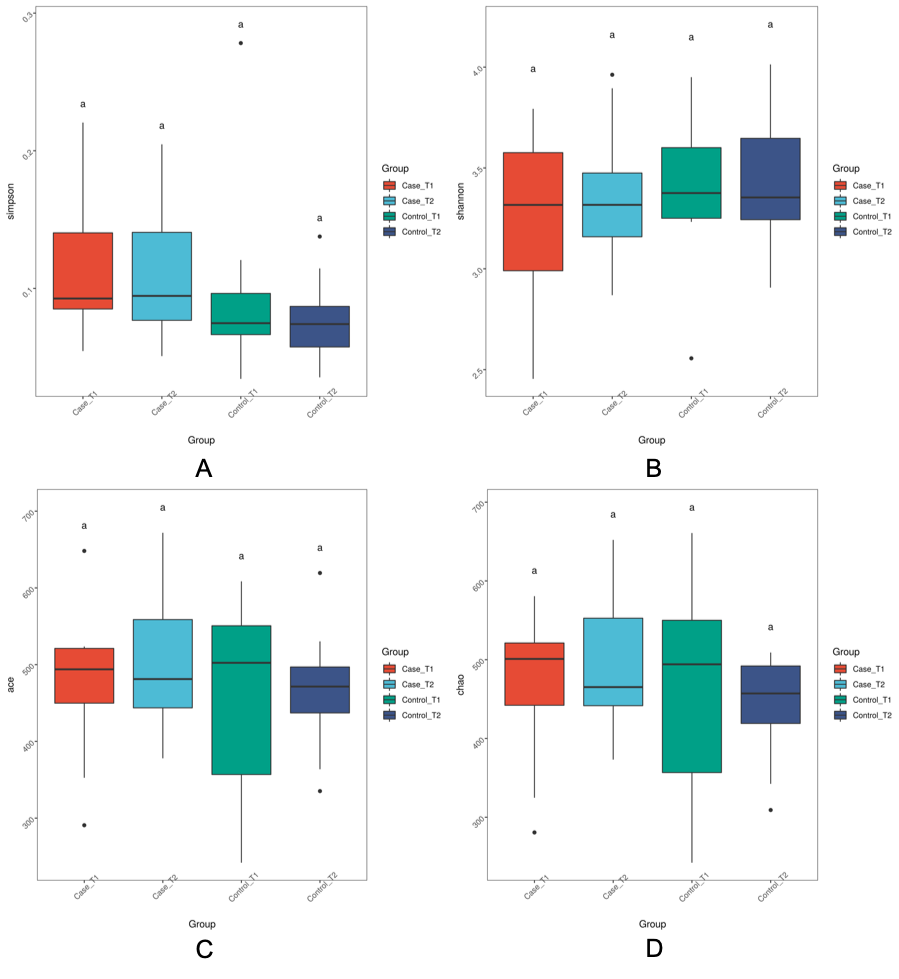


**Supplementary Figure S3.** Principal co-ordinates analysis program

A: Comparison before and after intervention in the intervention group; B: comparison before and after intervention in the control group


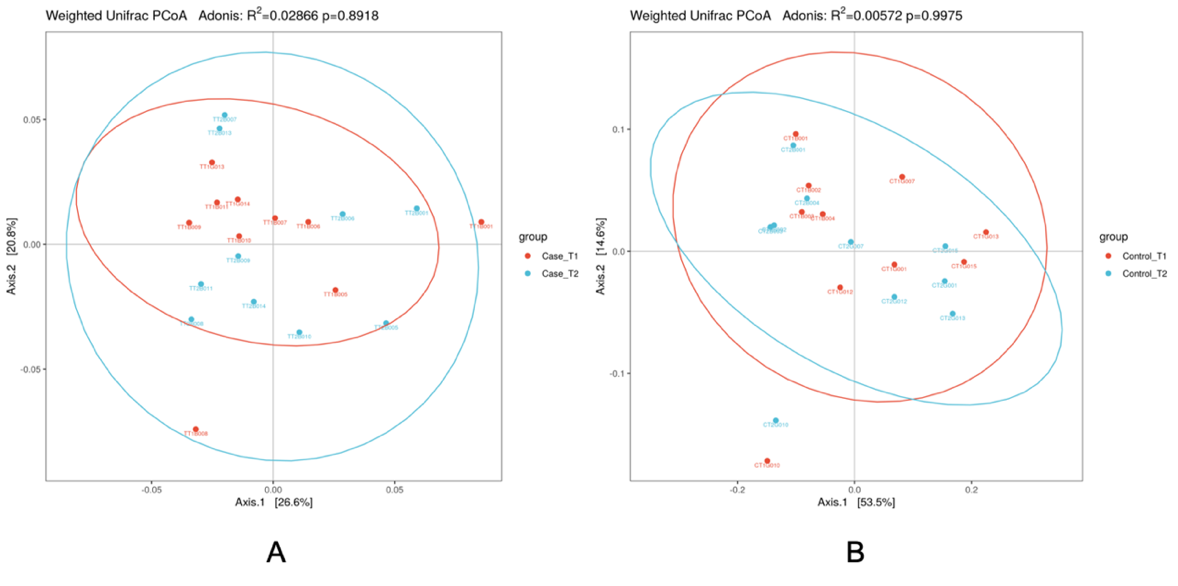


**Supplementary Figure S4.** Heat map

A: Comparison before and after intervention in the intervention group; B: comparison before and after intervention in the control group

**
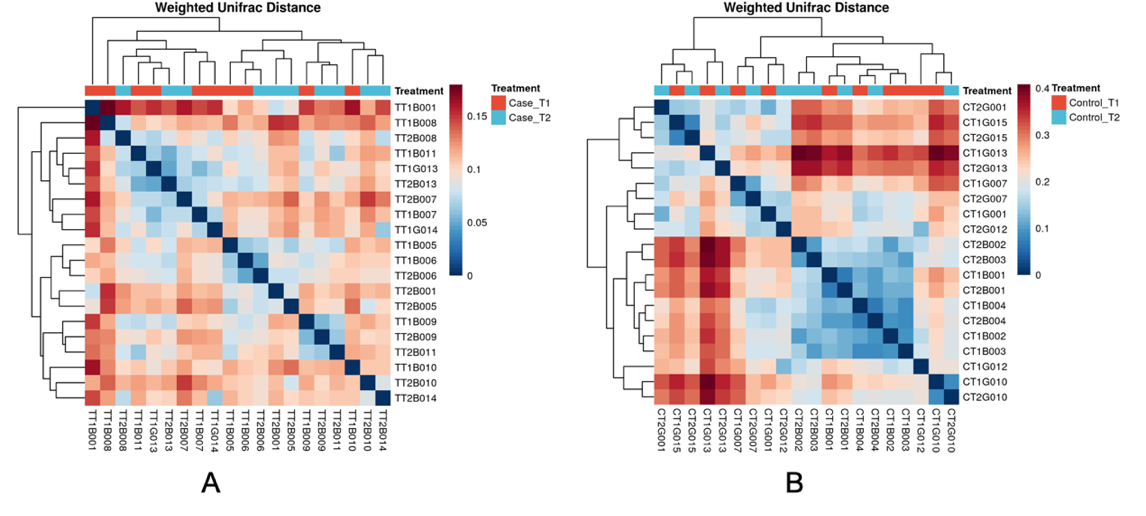
**

**Supplementary Figure S5.** LEfSe analysis results of the intervention group

A: Evolutionary branching diagram of LEfSe analysis; B: Histogram of LDA value distribution

**
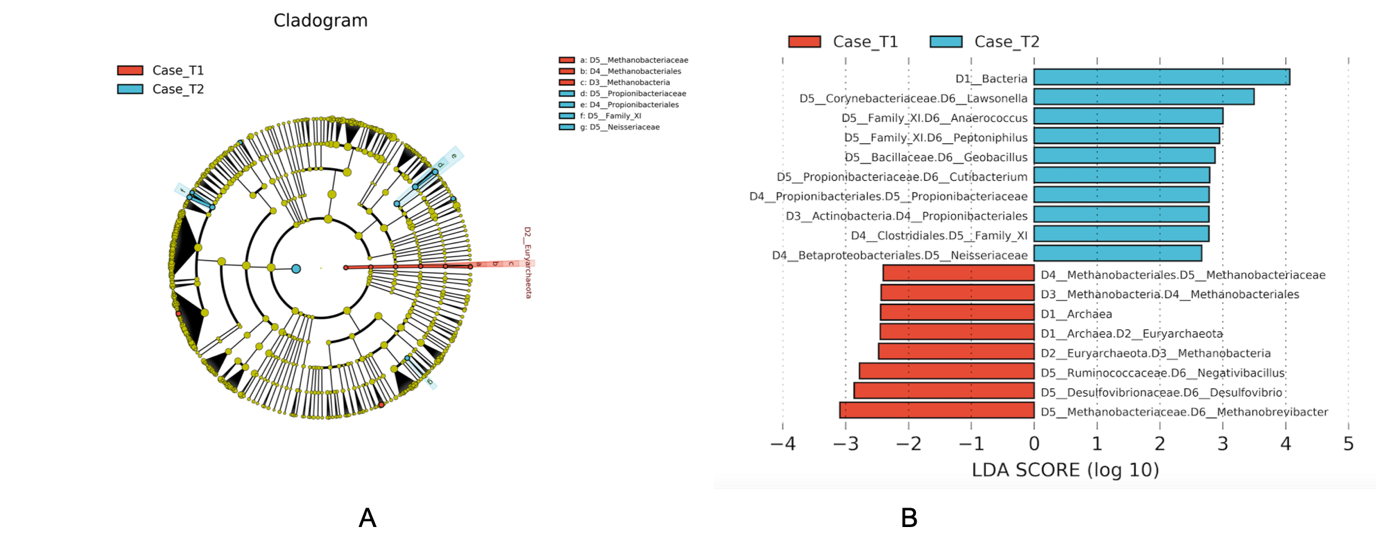
**

**Supplementary Figure S6.** LEfSe analysis results of the control group

A: Evolutionary branching diagram of LEfSe analysis; B: Histogram of LDA value distribution

**
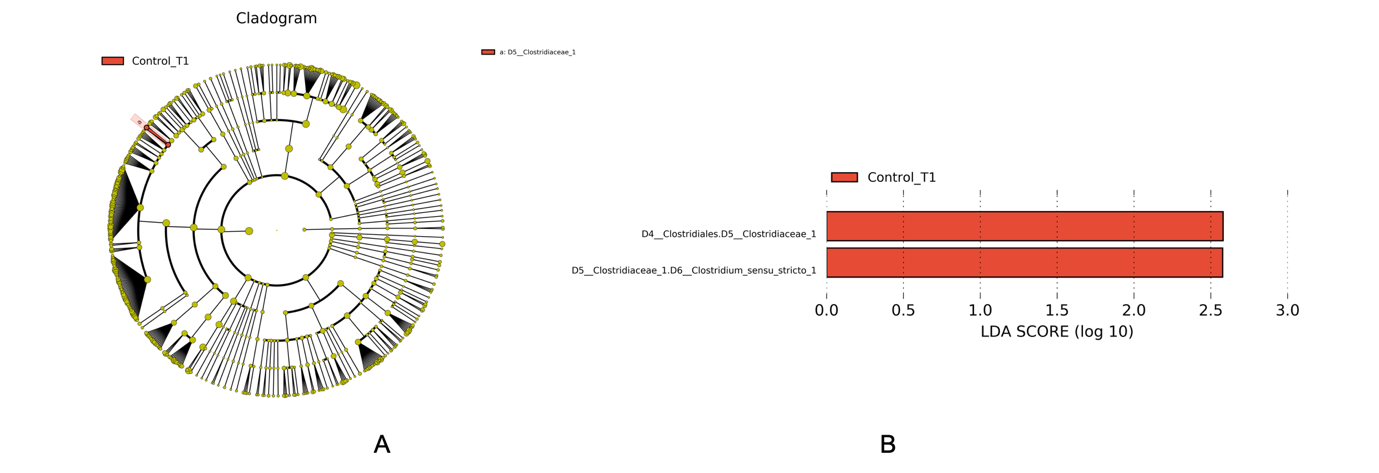
**
